# New generation sequencing of targeted genes in the classical and the variant form of hairy cell leukemia highlights mutations in epigenetic regulation genes

**DOI:** 10.18632/oncotarget.25601

**Published:** 2018-06-22

**Authors:** Elsa Maitre, Philippe Bertrand, Catherine Maingonnat, Pierre-Julien Viailly, Margaux Wiber, Dina Naguib, Véronique Salaün, Edouard Cornet, Gandhi Damaj, Brigitte Sola, Fabrice Jardin, Xavier Troussard

**Affiliations:** ^1^ Normandie Univ, INSERM U1245, Université de Caen, Caen, France; ^2^ Normandie Univ, INSERM U1245, Université de Rouen, Rouen, France; ^3^ Laboratoire d'hématologie, CHU Caen, Caen, France; ^4^ Service d'hématologie, Centre Henri Becquerel, Rouen, France; ^5^ Institut d'Hématologie de Basse-Normandie, CHU Caen, Caen, France

**Keywords:** hairy cell leukemia, next-generation sequencing, gene mutation, hairy cell leukemia variant, epigenetic regulation genes

## Abstract

Classical hairy cell leukemia (HCL-c) is a rare lymphoid neoplasm. *BRAF^V600E^* mutation, detected in more than 80% of the cases, is described as a driver mutation, but additional genetic abnormalities appear to be necessary for the disease progression. For cases of HCL-c harboring a wild-type *BRAF* gene, the differential diagnosis of the variant form of HCL (HCL-v) or splenic diffuse red pulp lymphoma (SDRPL) is complex. We selected a panel of 21 relevant genes based on a literature review of whole exome sequencing studies (*BRAF*, *MAP2K1*, *DUSP2*, *MAPK15*, *ARID1A*, *ARID1B*, *EZH2*, *KDM6A*, *CREBBP*, *TP53*, *CDKN1B*, *XPO1*, *KLF2*, *CXCR4*, *NOTH1*, *NOTCH2*, *MYD88*, *ANXA1*, *U2AF1*, *BCOR*, and *ABCA8*). We analyzed 20 HCL-c and 4 HCL-v patients. The analysis of diagnostic samples mutations in *BRAF* (*n* = 18), *KLF2* (*n* = 4), *MAP2K1* (*n* = 3), *KDM6A* (*n* = 2), *CDKN1B* (*n* = 2), *ARID1A* (*n* = 2), *CREBBP* (*n* = 2) *NOTCH1* (*n* = 1) and *ARID1B* (*n* = 1). *BRAF^V600E^* was found in 90% (18/20) of HCL-c patients. In HCL-c patients with *BRAF^V600E^*, other mutations were found in 33% (6/18) of cases. All 4 HCL-v patients had mutations in epigenetic regulatory genes: *KDM6A* (*n* = 2), *CREBBP* (*n* = 1) or *ARID1A* (*n* = 1). The analysis of sequential samples (at diagnosis and relapse) from 5 patients (2 HCL-c and 3 HCL-v), showed the presence of 2 new subclonal mutations (*BCOR^E1430X^* and *XPO1^E571K^*) in one patient and variations of the mutated allele frequency in 2 other cases. In the HCL-v disease, we described new mutations targeting *KDM6A* that encode a lysine demethylase protein. This opens new perspectives for personalized medicine for this group of patients.

## INTRODUCTION

Classical Hairy cell leukemia (HCL-c) is a rare lymphoid neoplasm with an incidence rate estimated at 0.3 per 100,000 people in United States between 2011 and 2012, and 0.2 per 100,000 people for the variant form of Hairy cell leukemia (HCL-v) [1]. The diagnosis of HCL-c is based on the typical hairy morphology of lymphocytes and the identification of *BRAF^V600E^* mutation [2–4]. The *BRAF^V600E^* mutation has been described as a driver mutation and detected in the hematopoietic stem cells (HSC) from HCL-c patients [5]. However, additional genetic abnormalities seem to be necessary to induce the disease. Tumor hairy cells display additional genetic alterations that are absent in HSC harboring *BRAF^V600E^*. Whole-exome sequencing (WES) studies have highlighted additional *BRAF^V600E^* single nucleotide variants (SNV) [4, 6–8]. Some of these SNVs are probably passenger mutations, but others, because of their recurrence, appear to be more relevant to the disease.

The *BRAF^V600E^* mutation is absent in some HCL-c patients [9, 10]. In these cases, the distinction between HCL-c and the variant form of hairy cell leukemia (HCL-v) or splenic diffuse red pulp lymphoma (SRDPL) can be complex. In HCL-c patients, the absence of *BRAF^V600E^* has been associated with an unmutated immunoglobulin heavy chain variable (IGHV) gene, the preferential use of the VH4-34 gene (7–11% of cases), mutations targeting *MAP2K1* (6/7 cases) and a poor prognosis [6, 10, 11]. HCL-v was introduced as provisional entity in the WHO classification of tumors in 2008 and 2016 [2, 3]. There are common characteristics between HCL-c and HCL-v, such as circulating villous lymphocytes and a histologic infiltration of the spleen with the involvement of the red pulp and atrophy of the white pulp [12]. The essential difference between the two diseases is the five-year overall survival rate: 78–92% for HCL-c and 57% for HCL-v [1, 13]. HCL-v diagnosis is essential because guidelines of care-management between HCL-c and HCL-v are different [14, 15]. The distinction between HCL-c and HCL-v is based on cytology with the presence of constant prominent nucleoli in HCL-v cells, as well as the immunophenotype. Classical hairy cells co-express CD103, CD123, CD25 and CD11c with an HCL score (one point given for each positive markers) ≥3 in 98% of cases [16]. The variant form lacks CD25 and has an HCL score <3. SDRPL, classified as a provisional entity by the WHO in 2008 and 2016, is a splenic hairy cell proliferation that is similar to HCL but quite distinct from splenic marginal zone lymphoma (SMZL) [17–19]. The distinction between HCL-v and SDRPL can be challenging because of an overlap in pathologies of the two cancers.

The next generation sequencing (NGS) approach and the sequencing of targeted genes have already proven to be useful for the diagnosis, classification and prognosis of lymphoid neoplasms [20]. Therefore, in the aim to develop a genomic diagnostic tool to distinguish these entities, we designed a panel of relevant target genes for HCL based on a literature review of WES studies [4, 6–8] and analyzed retrospectively HCL-c and HCL-v well-defined cases.

## RESULTS

### Single nucleotide variants (SNVs) featured in diagnosis samples

**Figure 1:**
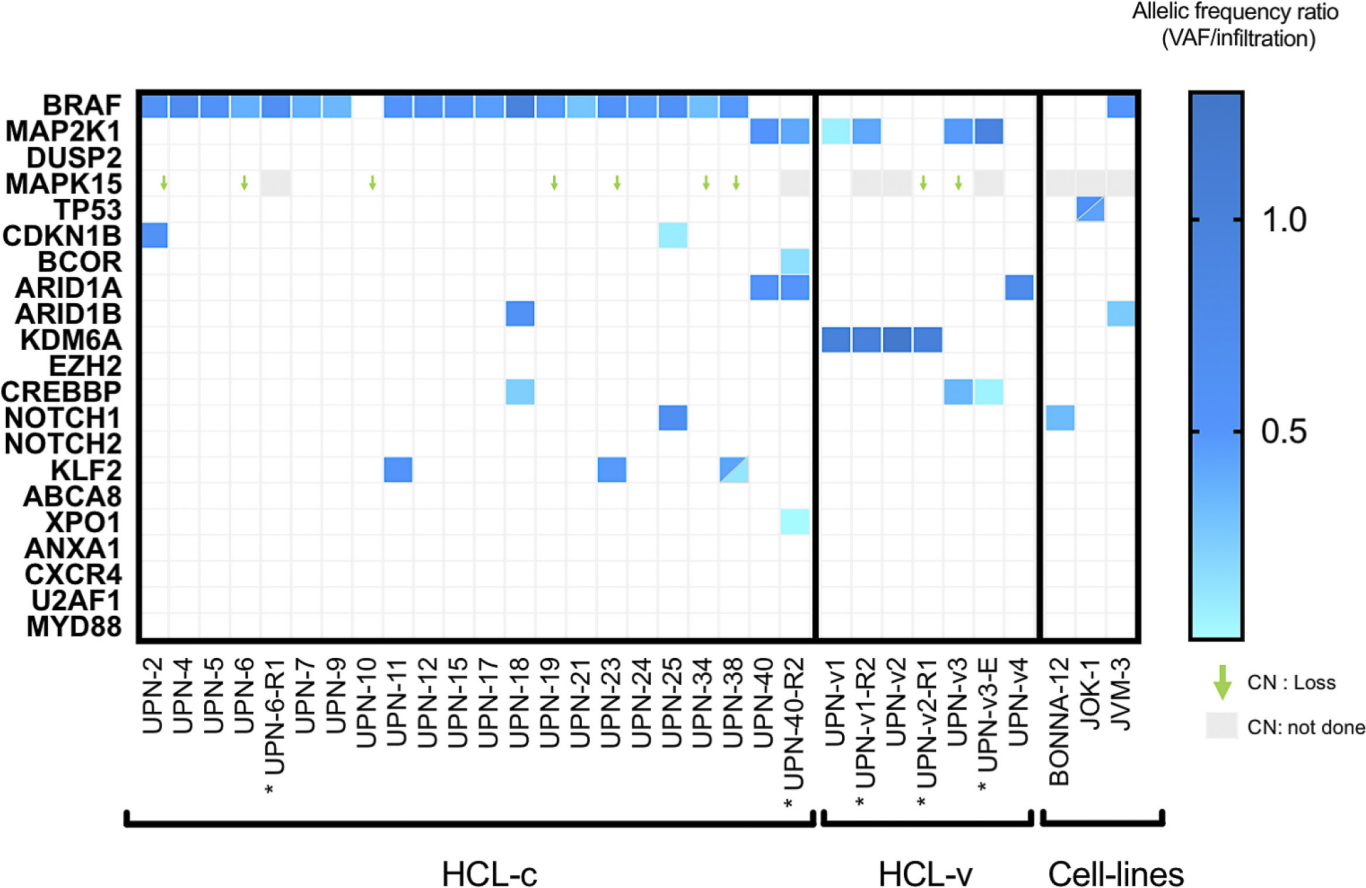
Heat-map representation of mutations and CNV distribution in HCL-c and HCL-v Each column represents one patient (UPN), and each row is one gene of the Trichopanel. The intensity of color is proportional to the rVAF (dark blue, rVAF value close to 1; light blue, rVAF value < 0.3). Gray represents relapse/evolution samples. Mutations and rVAF details are provided in the Supplementary Table 4. The gene copy number variation was reported for the relevant gene *MAPK15,* CN not done in gray. Abbreviations: CN, copy number; HCL-c, hairy cell leukemia classic form; HCL-v, variant form. ^*^ Relapse/evolution samples.

The Trichopanel was relevant for 96% (23/24) of patients. No SNVs were found in one patient (UPN-10) for any of the 21 targeted genes. The Trichopanel library sequencing yielded a median overall depth per sample of 322X [89X-533X] with 97.25% of targeted bases covered by 20 or more reads (Supplementary Table 2). For the samples obtained at diagnosis, a total of 891 variants (median per sample 37.5 [6–52]) were detected, then filtered on quality. Synonymous variations, intronic variations, and small nucleotide polymorphism (SNP) were excluded. Functional relevance was analyzed *in silico* using three validated algorithms (SIFT^®^, CADD^®^ and polyphen2^®^) (Supplementary Table 5). After screening, 35 non-synonymous SNVs were validated with a range of 0–3 variants per samples (Figure 1 and Supplementary Table 5). Finally, the Trichopanel identified relevant mutations in *BRAF* (*n* = 18), *KLF2* (*n* = 4), *MAP2K1* (*n* = 3), *KDM6A* (*n* = 2), *CDKN1B* (*n* = 2), *ARID1A* (*n* = 2), *CREBBP* (*n* = 2), *NOTCH1* (*n* = 1) and ARID1B (*n* = 1) (Figure 1).

### Cell lines and patient characteristics

The Trichopanel was validated by the sequencing of three cell lines (BONNA-12, JOK-1 and JVM-3). In agreement with previously published data, we found no *BRAF^V600E^* mutations in these cell lines, but other mutations were found (e.g., *NOTCH1^T2466M^* in BONNA-12, *BRAF^K601N^* in JVM-3 [21]) (COSMIC Cell lines project http://cancer.sanger.ac.uk/cell_lines). In addition, several new SNVs were found: *TP53^Y234H^*/*TP53^R213Q^* in JOK-1 and *ARID1B^P1435L^* in JVM-3. (Figure 1, Supplementary Table 4).

Patient characteristics of our cohort are summarized in Table 1 and in Supplementary Table 1. In total, 24 patients were analyzed (20 HCL-c and four HCL-v), and all patients provided samples at the time of diagnosis. For 5 patients, samples taken at the time of diagnosis and relapse/follow-up were investigated. In HCL-c patients, 88.2% (15/17) were treated with the first-line drug cladribine (*n* = 12), interferon a (*n* = 2) or pentostatine (*n* = 1). Forty percent (6/15) of those patients progressed with a median of 25 months [0–166.5]. Of the two untreated patients, one had no treatment criteria (UPN-19, 38.5 months of follow up), and the other (UPN-17) died on the day of the diagnosis.

**Table 1:** Patient characteristics of the cohort

*n* = patients	HCL-c (*n* = 20)	HCL-v (*n* = 4)	TOTAL (*n* = 24)
Diagnosis samples	20	4	24
Relapse/Evolution samples	2	3	5
Diagnosis age (years) (Median [min-max])	54.5 [42–92]	69.5 [64–82]	60.5 [42–92]
Sex Ratio Male/Female	15/5	4/0	19/5
Folow up (month) (Median [min-max])	37.1 [0.0–218.5]	19.9 [8.9–31.4]	32.3 [0.0–218.5]
Neutropenia (%, *n*)	82.3% (14/17)	0% (0/4)	66.7% (14/21)
Monocytopenia (%, *n*)	87.5% (14/16)	0% (0/4)	70% (14/20)
Anemia (%, *n*)	47% (8/17)	75% (3/4)	52.4% (11/21)
Treatment (%, *n*)	88.2% (15/17)	50% (2/4)	82.8% (18/22)
TFS (month) (Median [min-max])	1.4 [0.0–30.0]	6.05 [2.0–19.87]	1.7 [0.0–30.0]
Relapses (%, *n*)	40.0% (6/15)	50% (2/4)	44.4% (8/18)
PFS (month) (Median [min-max])	25.0 [0.0–166.5]	15.1 [4.1–26.0]	25.0 [0.0–166.5]

Definitions: Neutropenia: absolute polynuclear cells count (PNN) <1.5 × 10^9^/L; Monocytopenia: absolute monocytes count <0.20 × 10^9^/L; Anemia: hemoglobinemia rate <120 g/L (female) <130 g/L (male).

Abbreviations: PFS: Progression Free Survival, TFS: Treatment Free Suvival.

Of the HCL-v patients, two (UPN-v1 and UPN-v2) were treated with Rituximab plus cladribine or cladribine alone; they progressed rapidly at 4.1 and 26 months respectively. The remaining HLC-v patients (UPN-v3 and UPN-v4) were untreated because of palliative care and the absence of treatment criteria, respectively (follow up of 8.5 months).

### HCL-c patients at diagnosis have recurrent gene mutations

**Figure 2:**
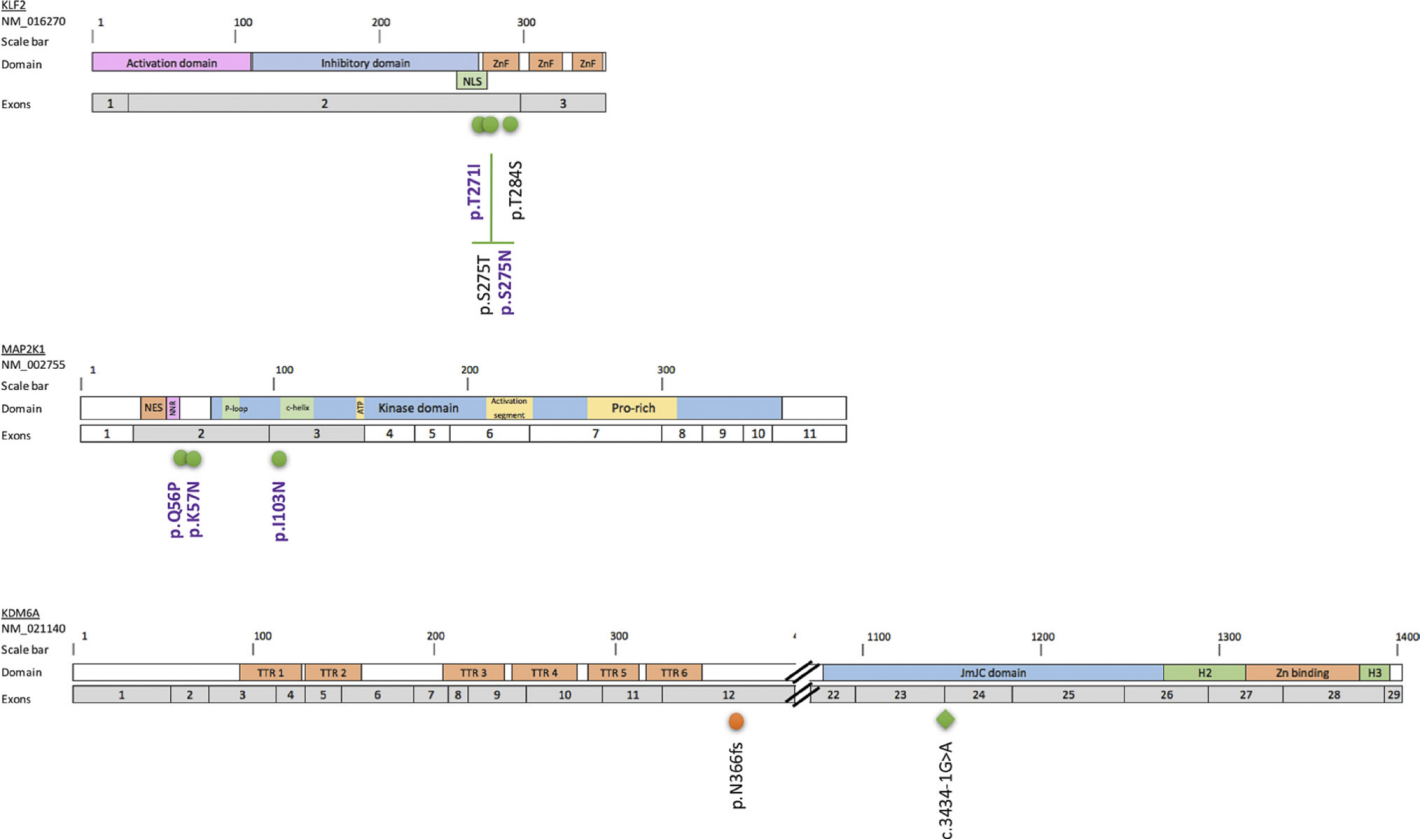
Localization of the *KLF2*, *MAP2K1* and *KDM6A* mutations in the corresponding proteins Distribution of mutations along the KLF2, MAP2K1 and KDM6A proteins. The exons targeted by the Trichopanel are represented in gray. Mutations are indicated as follows: green circles, missense mutations; blue circles, non-sense mutations; orange circles, deletions; green diamond, splicing variant. The SNVs previously described in HCL [8] or SMZL [25, 26] are in violet. Abbreviations: Helical domain (H), Jumonji domain (JmJC), nuclear export signal (NES), nuclear localization signal (NLS), negative regulatory region (NNR), Prolin-rich domain (Pro-rich), tetratricopeptids repeats (TTR), Zinc binding domain(Zn), Zinc fingers domain (ZnF).

The *BRAF^V600E^* mutation was found in 90% (18/20) of HCL-c patients. For the two patients without the *BRAF^V600E^* mutation, one (UPN-40) had a mutation in *MAP2K1,* and the other (UPN-10) had no alternative mutations in any of the targeted genes. The frequency of the *BRAF^V600E^* allele was compatible with heterozygosity, with a median rVAF of 0.48 ± 0.12 (Figure 1 and Supplementary Table 5). In addition to *BRAF^V600E^*, mutations in *KLF2* (*n* = 3), *CDKN1B* (*n* = 2), *NOTCH1* (*n* = 1), ARID1B (*n* = 1) and CREBBP (*n* = 1) were found in 33% (6/18) of cases. *KLF2* missense mutations found in three patients were localized in two specific domains, the zinc finger domain and the nuclear localization signal (Figure 2). *CDKN1B* mutations were found in two patients; these mutations were stop-gain and stop-loss mutations. One mutation had a rVAF compatible with heterozygosity (UPN-2; rVAF = 0.58), and the other was consistent with a sub-clonal mutation (UPN-25; rVAF = 0.09) (Supplementary Table 5).

### Epigenetic mutations are recurrent in HCL-v patients

The *MAP2K1^I103N^* and *MAP2K1^Q56P^* mutations were found in two of four HCL-v patients (UPN-v1 and UPN-v3, respectively). All HCL-v patients had SNVs in epigenetic regulatory genes including *KDM6A* (*n* = 2), *CREBBP* (*n* = 1) and *ARID1A* (*n* = 1) (Figure 1 and Supplementary Table 5). The two *KDM6A* mutations were potentially deleterious (UPN-v1 and UPN-v2). One was a frameshift deletion, and the other was a splicing variant leading to a loss of exon 24 as confirmed by RNA sequencing (Figure 2 and Supplementary Figure 1B). The calculated rVAF were 1.01 and 0.99, respectively, in these two male patients, in agreement with the location of *KDM6A* in the X chromosome.

### Sub-clonal mutations and variant allele frequency changes are found at the disease relapse

**Figure 3:**
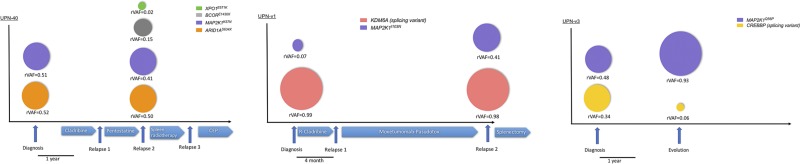
The mutations described at diagnosis are either maintained or evolving at relapse Representation of the relevant HCL-c (UPN-40) and HCL-v patients (UPNv1 and UPN-v3) tested at diagnosis and at relapse. For each patient, color-coded circles estimate the rVAF of the indicated mutations. Relevant times points and treatment are indicated by arrows. Abbreviations: CEP Cisplatin-Endoxan-Epirubicin, R-Cladribine: Rituximab + cladribine.

We analyzed serial samples from five patients (two HCL-c and three HCL-v) at diagnosis and relapse (Figure 3). For one HCL-c patient (UPN-6), we observed the same mutational pattern in the two samples. In the second case (UPN-40), two new sub-clonal mutations (*BCOR^E1430X^* and *XPO1^E571K^*) were characterized. In the three HCL-v patients (two treated and one untreated), no new mutations were observed but variations of the rVAF were identified in two cases. UPN-v1 had an increased allele frequency of *MAP2K1^I103N^* (rVAF = 0.07 at diagnosis and 0.41 at relapse). UPN-v3 had a decreased allele frequency of the *CREBBP* splicing variant (rVAF = 0.34 to 0.06). This patient was left untreated between the first and the second samples.

### *MAPK15* gene loss is the most frequent abnormality of CNVs

**Figure 4:**
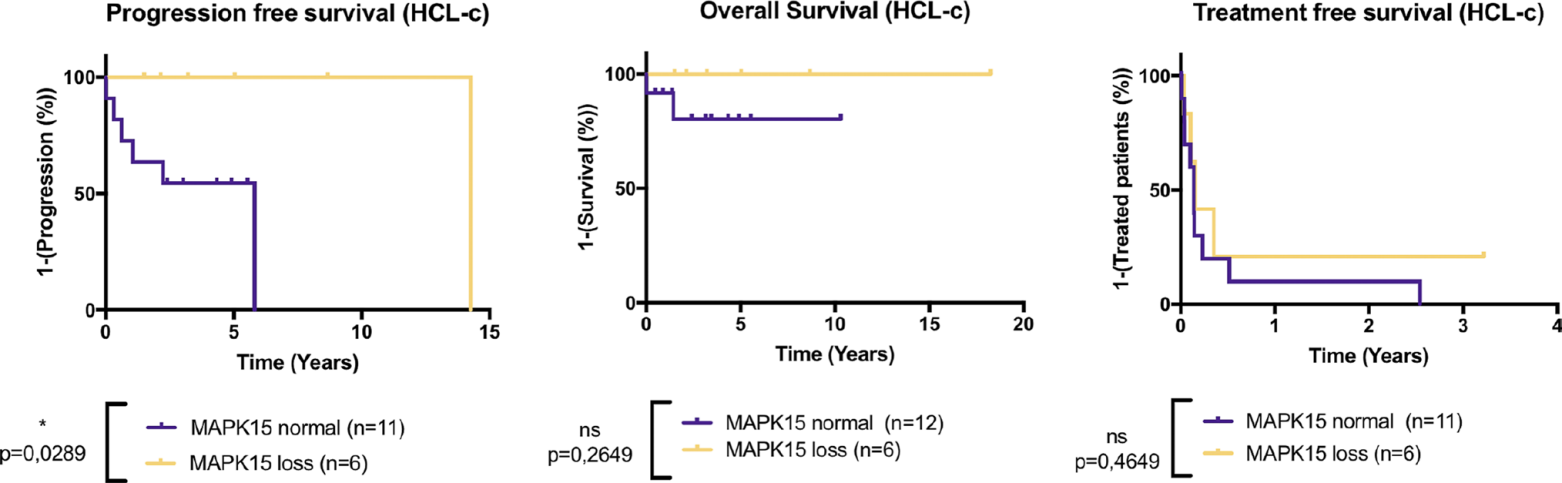
*MAPK15* deletion improves the progression-free survival in HCL-c patients Kaplan-Meier representation of Treatment-free survival (TFS), Progression-free survival (PFS) and Overall survival (OS) of HCL-c according to *MAPK15* copy loss status. *p*-values were calculated with Log-rank Test. In the “*MAPK15* normal” group, patients were given first-line treatment with Interferon *n* = 1, pentostatine *n* = 2 or cladribine *n* = 5. In the “*MAPK15* loss” group, patients were given first-line treatment with Interferon *n* = 1 or cladribine *n* = 6. *MAPK15* loss is associated with significantly improved PFS in HLC-c patients, and no significant differences were observed in TFS and OS.

CNV analysis highlighted various abnormalities: the most frequent CNV was a loss of one copy of *MAPK15* found in 37.5% of patients (9/24) (Figure 1). According to the *MAPK15* CNV status, there were no significant differences for TFS and OS in HCL-c patients, but PFS was significantly better in HCL-c patients with a *MAPK15* deletion (Figure 4). Four patients had a loss of *EZH2* gene copy (UPN-18, UPN-40, UPN-v1 and UPN-v2). On the basis of their karyotypes, two of them had an abnormal chromosome 7q (one deletion and one rearrangement) involving the loss of *BRAF* (7q34) (Supplementary Table 1).

## DISCUSSION

HCL cell lines are atypical. As expected, none had the *BRAF^V600E^* mutation [23], suggesting that a hairy cell phenotype-like can be observed without this driver mutation. In contrast, other mutations were found in *TP53*, *NOTCH1*, *ARID1B* and *BRAF* with an allele frequency compatible with their complex karyotype (Supplementary Table 4). JVM-3 is a B prolymphocytic leukemia-derived cell line and harbors *BRAF^K601N^* [22]. This mutation is found in rare cases of melanoma and leads to MAPK pathway activation [24]. Altogether, the cell line data show that JVM-3 could be a model for studying HCL.

Consistent with the literature, *BRAF^V600E^* mutations were found in 90% (18/20) of HCL-c patients [4, 10]. The proportion of HCL-c patients with *BRAF^WT^* differs according to the studies, ranging from 0 to 20% [4, 10]. Some alternative *BRAF^V600E^* mutations were found in exon 11 [9]. In this study, the two HCL-c patients with *BRAF*^WT^ had no alternative BRAF mutations in exons 11 or 15. For these patients, the diagnosis of the classical form was confirmed by immunophenotyping, the HCL score (4/4), and the hairy cell morphology without prominent nucleoli. These criteria indicate that *BRAF^V600E^* is not the unique driver mutation in all HCL-c cases. IGHV-4-34 gene rearrangements are associated with *BRAF^WT^* in HCL-c patients [10]. In this study, IGHV status was not examined.

One third of the HCL-c patients had mutations in addition to *BRAF* mutations (one to two additional altered genes per patient). In all patients with these additional mutations (6/6), rVAFs were close to 0.50 (0.47 ± 0.16), confirming the early-onset of the mutation and the hypothesis in which mutations in addition to *BRAF* are necessary to the disease [5]. Two HCL-c patients with *BRAF^V600E^* (UPN-25 and UPN-38) had additional sub-clonal mutations in *CDKN1B^W76X^* (rVAF = 0.09) and *KLF2^T284S^* (rVAF = 0.16), respectively. The characterization of these sub-clonal mutations could be necessary for the management of future treatment and to avoid the promotion of a sub-clone during the relapse.

The Trichopanel detected *KLF2* mutations in 15% (3/20) of HCL-c patients. This percentage is comparable with those reported in the literature, reported in 10–16% of cases [25, 26]. *KLF2*, involved in three patients, plays a key role in B-cell homing to lymph nodes and inhibition of the NF-κB pathway^26,27^. *KLF2^275N^*, *KLF2^275T^* and *KFL2^T271I^* mutations found in those patients are localized within the zinc finger domain or the nuclear localization signal (NLS). These mutations have been described in splenic zone marginal lymphoma (SMZL) as possibly deleterious mutations [25, 26]. Moreover, mutations involving the NLS lead to cytoplasmic relocalization of KFL2 and affect transcription factor activity [26]. In a murine model, KLF2 gene knock-out is not sufficient to induce lymphoma but results in deregulation of B-cell differentiation and trafficking [27, 28]. Thus, mutations in *KLF2* in HCL-c patients could explain both the extra-nodal localization of hairy cells and NF-κB pathway upregulation [29]. One patient (UPN-38) had two mutations in KLF2: *KFL2^T271I^* (rVAF = 0.42) and *KLF2^T284S^* (rVAF = 0.16). Multiple KLF2 mutations (2–5) have been described previously in eleven patients with HLC-c (*n* = 1), extra-nodal marginal zone lymphoma (=1), Burkitt lymphoma (*n* = 1), SMZL (*n* = 1) and diffuse large B cell lymphoma (*n* = 7), and in most cases, the *KLF2* mutations were located on separated alleles [26]. The effects of *KLF2* mutations on HCL are still unclear and further studies are necessary.

Alterations of cell cycle and apoptosis are common in hematologic neoplasms. In this study, two HCL-c patients had *CDKN1B* mutations (2/20, 10%), and none had *TP53* mutations. These data are consistent with previously published data in which *CDKN1B* mutations are found in 11–16% of HCL-c patients [7, 30]. In HCL-c patients, the frequency of *TP53* mutations differs considerably from one study to another, ranging from 0–2% [30, 31] to 27% [32]. In our study, patients with *CDKN1B^W76X^* and *CDKN1B^X199S^* mutations had an atypical HCL immunophenotype with the expression of CD10. CD10 expression in HCL is rare (10–20% of cases) [33]. Currently, no studies have reported a correlation between *CDKN1B* mutation and CD10 expression.

In three of the six patients with *BRAF^WT^* (2 HCL-c and 4 HCL-v), we found mutations in *MAP2K1*. This finding is consistent with previously published data in which 48% of HCL (classical and variant form) patients with *BRAF^WT^* had MAP2K1 mutations [6]. More precisely, *MAP2K1* activating mutations are found in 86% (6/7) of *BRAF^WT^-*containing HCL-c patients and 42% (10/24) of HCL-v patients [6]. *BRAF* and *MAP2K1* mutations are mutually exclusive in HCL [6]. The mutations *MAP2K1^Q56P^* and *MAP2K1^K57N^* (UPN-v3 and UPN-40, respectively) that we found are close to the negative regulatory domain of the protein and lead to ERK phosphorylation [34, 35]. Interestingly, although the presence of *MAP2K1^K57N^* would improve of the sensitivity of cells to MEK inhibitors, *MAP2K1^Q56P^* could generate resistance [35, 36]. *MAP2K1^I103N^* (in UPN-v1) is localized close to the C-terminal helix of the protein [34]. Currently, this mutation is not described as an MEK activator but could confer resistance to MEK inhibitors by impairing the allosteric binding of the drug [35]. In the UPN-v1 patient, the *MAP2K1^I103N^* mutation was sub-clonal at the initial diagnosis (rVAF = 0.07) and considerably increased at the time of relapse (rVAF = 0.41). Altogether, these data showed that *MAP2K1^I103N^* would not confer a proliferative or survival advantage at time of diagnosis but could induce a bad-responder phenotype, even in the absence of MEK inhibitor. Indeed, UPN-v1 was treated with cladribine-rituximab and then moxetumomab-pasudotox as a second line of treatment. The detection of these mutations seems essential in the future clinical management of HCL patients.

No *TP53* mutations were found in HCL-v patients, even at relapse. *TP53* mutations have been described in nearly 30% of HCL-v cases [13, 31].

Finally, we described new *KDM6A* mutations in HCL-v patients. Both patients were hemizygous for the mutations as *KDM6A* is located on chromosome X. The presence of tumor suppressor genes on the chromosome X has been used to explain the imbalanced male/female sex ratio in cancers [37]. Moreover, HCL is known to have a 5:1 male/female sex ratio [3]. *KDM6A* (also known as Ubiquitously Transcribed Tetratricopeptide Repeat Protein X-Linked (UTX)) encodes a lysine demethylase protein that removes di- and tri-methyl groups from lysine 27 of Histone 3 (H3K27). Disruptive *KDM6A* mutations have been found in multiple myeloma, bladder neoplasms and T cell acute lymphoblastic leukemia (T-ALL) [38]*. KDM6A* mutations leading to a loss of function were previously described in 3 HCL-c patients [6, 7, 39]. Two were atypical patients, and one had a stop-gain mutation in *KDM6A* found in the relapse sample, possibly due to vemurafenib treatment [7]. Another patient had the *BRAF*^WT^ allele with an unmutated immunoglobulin heavy gene VH4-34 [6]. This sub-type of HCL is considered to be bad responder to classical drug therapies [31]. A disruptive *KDM6A* mutation was also recently found in one SDRPL patient and in one HCL-v patient [30, 39]. In our series, one patient with a *KDM6A* mutation was non-responsive to first and second line therapies.

Those mutations and the two mutations found in our series result in the loss of the highly conserved C-terminal region of KDM6A (including Jumomji and zinc binding domains) which is essential for its demethylase activity [40]. Loss of KDM6A activity may sensitize tumor cells to demethylating agents such as EZH2 inhibitors [41]. Indeed, KDM6A inactivation (*KDM6A^R1279X^*) in T-ALL cell lines showed an improvement in the sensitivity to DZNEP, an epigenetic compound that targets methylation of H3K27 [42].

As already described in chronic lymphocytic leukemia [43], the analysis of diagnosis/relapse samples highlighted the heterogeneity of tumor cells and their sub-clonal evolution during the course of the disease. *BCOR* was recently described as a new recurrent gene altered in SDRPL. Mutations were found in 6/42 SRDPL and loss of *BCOR* expression in 4 other cases [39]. In our series, a *BCOR* mutation (*BCOR^E1430X^*) was found at the relapse of patient UPN-40 as a sub-clonal stop-gain mutation (rVAF = 0.15).

*XPO1* encodes for exportin 1, a protein that plays a key role in the nuclear export of tumor suppressors such as p53, p27 and Iκ-B, an inhibitor of the NF-κB pathway [44]. A hotspot *XPO1^E571K^* mutation has been found in primary mediastinal B-cell lymphoma, classical Hodgkin lymphoma (HL-c) and chronic lymphocytic leukemia (CLL) [45–47]. One patient (UPN-40) presented the *XPO1^E571K^* hotspot mutation at relapse. The significance of the presence of a sub-clonal *XPO1^E571K^* mutation at relapse is unknown.

The *MAPK15* gene encodes extracellular regulated kinase 8 (ERK8), a recently identified member of the MAPK family. This gene is located in chromosome 8. Its activity is still unclear, but it appears to play an oncogenic role. ERK8 stabilizes c-JUN and regulates autophagy and cell transformation [48, 49]. Overexpression of *MAPK15* was described in solid neoplasms [49, 50]. Thanks to this effect on MAPK pathway, *MAPK15* loss could potentially antagonize the constitutive activation of MAPK pathway in *BRAF^V600E^* hairy cells. Further investigations of the link between *BRAF^V600E^* and *MAPK15* are necessary. Here, we found that loss of *MAPK15* copy number improved PFS in HCL-c patients without repercussion on OS (Figure 4).

## MATERIALS AND METHODS

### Patients and patient samples

We studied three cell lines (BONNA-12, JOK-1, JVM3 (obtained from German Collection of Microorganisms and Cell Culture or offered by Toulouse University Hospital) 20 HCL-c and 4 HCL-v patients from an initial diagnosis and 2 HCL-c and 3 HCL-v patients with relapsing disease. Mononuclear cells were obtained from peripheral blood (17 samples), bone marrow aspirates (10 samples) or 2 biopsy specimens (spleen and nodule). The HCL-c disease was diagnosed in accordance with the WHO 2016 classification [3] by combination of clinical criteria, cytology, presence of *BRAF^V600E^* and immunophenotyping. The HCL-v disease was diagnosed by clinical criteria, hairy cell morphology with constant prominent nucleoli, the absence of *BRAF^V600E^*, and immunophenotyping (Supplementary Table 1). Informed consent was obtained from patients, and the procedures were conducted in accordance with the Helsinki Declaration and the policy of the CHU de Caen.

### Next generation sequencing

The PBMC fraction was collected after gradient density separation (histopaque^®^). DNA was extracted with the automated device MagnaPur^®^ (Roche Lifescience) according to the manufacturer's recommendations. Library design was performed with the Ion Ampliseq Designer™ software. The DNA library (Ion Ampliseq™ Library kit), template preparation/chip loading (Ion Chef™ system + Ion PGM™ Hi-Q Chef Kit Reagent) and sequencing (Ion Torrent PGM™) were performed according to the manufacturer's recommendations (ThermoFisher Scientific). The Trichopanel design covers 71,020 bases using 712 amplicons (Supplementary Table 2). The analyzed genes belong to nine functional groups: MAPK signaling pathway (*BRAF*, *MAP2K1*, *DUSP2*, *MAPK15*), epigenetic regulation (*ARID1A*, *ARID1B*, *EZH2*, *KDM6A*, *CREBBP*), cell cycle/apoptosis (*TP53*, *CDKN1B*, *XPO1*), homing (*KLF2*, *CXCR4*), NOTCH pathway (*NOTCH1*, *NOTCH2*), NF-κB pathway (*MYD88*), inflammation (*ANXA1*), splicing (*U2AF1*), differentiation (*BCOR*) and extracellular transport (*ABCA8*), according to published WES data [4, 6–8]. Data analysis was performed with Torrent suite^TM^ software, and then, variant analysis was performed using an in-house generated bioinformatic pipeline (Generate reports^®^) as previously described [20]. The ratio of variant allele frequency (VAF), rVAF, was calculated as the percentage of VAF on divided by the percentage of tumor infiltration. Splicing prediction was performed using an in-house generated bioinformatic pipeline (Alamut^®^ Visual 2.9 interactive Biosoftware) as previously described [51] (Supplementary Figure 1A). Functional relevance was analyzed *in silico* using three validated algorithms (SIFT^®^, CADD^®^ and polyphen2^®^).

Copy number variation (CNV) analysis of the regions sequenced by the Trichopanel was performed as previously described [52], and patients' data were normalized to DNA samples from eight healthy patients.

### Sanger sequencing

Primer sequences for PCR amplification were designed with the Primer3 software (v4.0.0, http://primer3.ut.ee) (Supplementary Table 3). Sequencing was performed on an ABI Prism31000 device (ThermoFisher Scientific) according to the manufacturer's recommendations.

### Immunophenotyping

Multiparameter flow cytometric immunophenotyping was performed on a FACS CANTO II or a FACSCalibur (Becton Dickinson, (BD)), and the data were used to characterize hairy cells (HC) and to quantify tumor infiltration (See Supplementary Methods).

### Statistical analysis

Statistical representations of the Kaplan–Meir test on Treatment Free Survival (TFS), Progression Free Survival (PFS) and Overall Survival (OS) were performed using GraphPad Prism version 7.00, and *p*-values were calculated with Log-rank Test. TFS was calculated from the date of diagnosis to date of first treatment or last patient follow-up. OS was calculated from the date of diagnosis to date of death or last patient follow-up. PFS was calculated from the date of diagnosis until disease progression, relapse, death or last patient follow-up. *p* values < 0.05 were considered statistically significant.

## CONCLUSIONS

The use of the Trichopanel has a potential benefit in the diagnosis and the prognosis of HCL-c and HCL-v patients, who need to be confirmed in a larger cohort. This is a relatively easy tool for routine analysis. The *KDM6A* loss of function mutation described in this study needs to be further investigated in order to determine the role of demethylating agents in those patients.

## SUPPLEMENTARY MATERIALS FIGURES AND TABLES









## Abbreviations

CNV: copy number variation
HCL-c: classical hairy cell leukemia
HCL-v: hairy cell leukemia-variant
SDRPL: splenic diffuse red pulp lymphoma
SMZL: splenic marginal zone lymphoma
SNV: single nucleotide variation

## References

[1] Teras LR, DeSantis CE, Cerhan JR, Morton LM, Jemal A, Flowers CR (2016). US Lymphoid Malignancy Statistics by World Health Organization Subtypes. CA Cancer J Clin.

[2] Swerdlow SH, Campo E, Lee Harris N, Jaffe ES, Pileri SA, Stein H, Thiele J, Vardiman JW (2008). WHO Classification of tumours of Haematopoietic and Lymphoid Tissues.

[3] Swerdlow SH, Campo E, Pileri SA, Harris NL, Stein H, Siebert R, Advani R, Ghielmini M, Salles GA, Zelenetz AD, Jaffe ES (2016). The 2016 revision of the World Health Organization classification of lymphoid neoplasms. Blood.

[4] Tiacci E, Trifonov V, Schiavoni G, Holmes A, Kern W, Martelli MP, Pucciarini A, Bigerna B, Pacini R, Wells VA, Sportoletti P, Pettirossi V, Mannucci R (2011). BRAF mutations in hairy-cell leukemia. N Engl J Med.

[5] Chung SS, Kim E, Park JH, Chung YR, Lito P, Teruya-Feldstein J, Hu W, Beguelin W, Monette S, Duy C, Rampal R, Telis L, Patel M (2014). Hematopoietic stem cell origin of BRAFV600E mutations in hairy cell leukemia. Sci Transl Med.

[6] Waterfall JJ, Arons E, Walker RL, Pineda M, Roth L, Killian JK, Abaan OD, Davis SR, Kreitman RJ, Meltzer PS (2013). High prevalence of MAP2K1 mutations in variant and IGHV4-34–expressing hairy-cell leukemias. Nat Genet.

[7] Dietrich S, Hüllein J, Lee SC, Hutter B, Gonzalez D, Jayne S, Dyer MJ, Oleś M, Else M, Liu X, Słabicki M, Wu B, Troussard X (2015). Recurrent CDKN1B (p27) mutations in hairy cell leukemia. Blood.

[8] Weston-Bell NJ, Tapper W, Gibson J, Bryant D, Moreno Y, John M, Ennis S, Kluin-Nelemans HC, Collins AR, Sahota SS, Richards KL (2016). Exome Sequencing in Classic Hairy Cell Leukaemia Reveals Widespread Variation in Acquired Somatic Mutations between Individual Tumours Apart from the Signature BRAF V(600)E Lesion. PLoS One.

[9] Tschernitz S, Flossbach L, Bonengel M, Roth S, Rosenwald A, Geissinger E (2014). Alternative BRAF mutations in BRAF V600E-negative hairy cell leukaemias. Br J Haematol.

[10] Xi L, Arons E, Navarro W, Calvo KR, Stetler-Stevenson M, Raffeld M, Kreitman RJ (2012). Both variant and IGHV4-34–expressing hairy cell leukemia lack the BRAF V600E mutation. Blood.

[11] Arons E, Kreitman RJ (2011). Molecular variant of hairy cell leukemia with poor prognosis. Leuk Lymphoma.

[12] Matutes E, Martínez-Trillos A, Campo E (2015). Hairy cell leukaemia-variant: Disease features and treatment. Best Pract Res Clin Haematol.

[13] Hockley SL, Else M, Morilla A, Wotherspoon A, Dearden C, Catovsky D, Gonzalez D, Matutes E (2012). The prognostic impact of clinical and molecular features in hairy cell leukaemia variant and splenic marginal zone lymphoma. Br J Haematol.

[14] Robak T, Matutes E, Catovsky D, Zinzani PL, Buske C (2015). Hairy cell leukaemia: ESMO Clinical Practice Guidelines for diagnosis, treatment and follow-up. Ann Oncol.

[15] Grever MR, Abdel-Wahab O, Andritsos LA, Banerji V, Barrientos J, Blachly JS, Call TG, Catovsky D, Dearden C, Demeter J, Else M, Forconi F, Gozzetti A (2017). Consensus guidelines for the diagnosis and management of patients with classic hairy cell leukemia. Blood.

[16] Matutes E, Morilla R, Owusu-Ankomah K, Houliham H, Meeus P, Catovsky D (1994). The immunophenotype of hairy cell leukemia (HCL). Proposal for a scoring system to distinguish HCL from B-cell disorders with hairy or villous lymphocytes. Leuk Lymphoma.

[17] Baseggio L, Traverse-Glehen A, Callet-Bauchu E, Morel D, Magaud JP, Berger F, Salles G, Felman P (2011). Relevance of a scoring system including CD11c expression in the identification of splenic diffuse red pulp small B-cell lymphoma (SRPL). Hematol Oncol.

[18] Traverse-Glehen A, Baseggio L, Callet-Bauchu E, Morel D, Gazzo S, Ffrench M, Verney A, Rolland D, Thieblemont C, Magaud JP, Salles G, Coiffier B, Berger F (2008). Splenic red pulp lymphoma with numerous basophilic villous lymphocytes: a distinct clinicopathologic and molecular entity?. Blood.

[19] Traverse-Glehen A, Verney A, Gazzo S, Jallades L, Chabane K, Hayette S, Coiffier B, Callet-Bauchu E, Ffrench M, Felman P, Berger F, Baseggio L, Salles G (2016). Splenic diffuse red pulp lymphoma has a distinct pattern of somatic mutations amongst B-cell malignancies. Leuk Lymphoma.

[20] Dubois S, Viailly PJ, Mareschal S, Bohers E, Bertrand P, Ruminy P, Maingonnat C, Jais JP, Peyrouze P, Figeac M, Molina TJ, Desmots F, Fest T (2016). Next Generation Sequencing in Diffuse Large B Cell Lymphoma Highlights Molecular Divergence and Therapeutic Opportunities: a LYSA Study. Clin Cancer Res.

[21] Nayak L, Goduni L, Takami Y, Sharma N, Kapil P, Jain MK, Mahabeleshwar GH (2013). Kruppel-Like Factor 2 Is a Transcriptional Regulator of Chronic and Acute Inflammation. Am J Pathol.

[22] Jebaraj BM, Kienle D, Bühler A, Winkler D, Döhner H, Stilgenbauer S, Zenz T (2013). BRAF mutations in chronic lymphocytic leukemia. Leuk Lymphoma.

[23] Tiacci E, Pucciarini A, Bigerna B, Pettirossi V, Strozzini F, Martelli MP, Tabarrini A, Drexler HG, Falini B (2012). Absence of BRAF-V600E in the human cell lines BONNA-12, ESKOL, HAIR-M, and HC-1 questions their origin from hairy cell leukemia. Blood.

[24] Yao Z, Torres NM, Tao A, Gao Y, Luo L, Li Q, de Stanchina E, Abdel-Wahab O, Solit DB, Poulikakos PI, Rosen N (2015). BRAF Mutants Evade ERK-Dependent Feedback by Different Mechanisms that Determine Their Sensitivity to Pharmacologic Inhibition. Cancer Cell.

[25] Clipson A, Wang M, de Leval L, Ashton-Key M, Wotherspoon A, Vassiliou G, Bolli N, Grove C, Moody S, Escudero-Ibarz L, Gundem G, Brugger K, Xue X (2015). KLF2 mutation is the most frequent somatic change in splenic marginal zone lymphoma and identifies a subset with distinct genotype. Leukemia.

[26] Piva R, Deaglio S, Famà R, Buonincontri R, Scarfò I, Bruscaggin A, Mereu E, Serra S, Spina V, Brusa D, Garaffo G, Monti S, Dal Bo M (2015). The Krüppel-like factor 2 transcription factor gene is recurrently mutated in splenic marginal zone lymphoma. Leukemia.

[27] Hart GT, Wang X, Hogquist KA, Jameson SC (2011). Kruppel-like factor 2 (KLF2) regulates B-cell reactivity, subset differentiation, and trafficking molecule expression. Proc Natl Acad Sci.

[28] Winkelmann R, Sandrock L, Porstner M, Roth E, Mathews M, Hobeika E, Reth M, Kahn ML, Schuh W, Jack HM (2011). B cell homeostasis and plasma cell homing controlled by Kruppel-like factor 2. Proc Natl Acad Sci.

[29] Nagel S, Ehrentraut S, Meyer C, Kaufmann M, Drexler HG, MacLeod RA (2015). NFkB is activated by multiple mechanisms in hairy cell leukemia: NFkB IS Activated by Multiple Mechanisms. Genes Chromosomes Cancer.

[30] Durham BH, Getta B, Dietrich S, Taylor J, Won H, Bogenberger JM, Scott S, Kim E, Chung YR, Chung SS, Hüllein J, Walther T, Wang L (2017). Genomic analysis of hairy cell leukemia identifies novel recurrent genetic alterations. Blood.

[31] Forconi F, Sozzi E, Cencini E, Zaja F, Intermesoli T, Stelitano C, Rigacci L, Gherlinzoni F, Cantaffa R, Baraldi A, Gallamini A, Zaccaria A, Pulsoni A (2009). Hairy cell leukemias with unmutated IGHV genes define the minor subset refractory to single-agent cladribine and with more aggressive behavior. Blood.

[32] König EA, Kusser WC, Day C, Porzsolt F, Glickman BW, Messer G, Schmid M, De Chatel R, Marcsek ZL, Demeter J (2000). p53 mutations in hairy cell leukemia. Leukemia.

[33] Jasionowski TM, Hartung L, Greenwood JH, Perkins SL, Bahler DW (2003). Analysis of CD10+ Hairy Cell Leukemia. Am J Clin Pathol.

[34] Bromberg-White JL, Andersen NJ, Duesbery NS (2012). MEK genomics in development and disease. Brief Funct Genomics.

[35] Emery CM, Vijayendrana KG, Zipserc MC, Sawyera AM, Niua L, Kima JJ, Hattona C, Choprad R, Oberholzera PA, Karpovac MB, MacConailla LE, Zhang J, Gray NS (2009). MEK1 mutations confer resistance to MEK and B-RAF inhibition. PNAS.

[36] Marks JL, Gong Y, Chitale D, Golas B, McLellan MD, Kasai Y, Ding L, Mardis ER, Wilson RK, Solit D, Levine R, Michel K, Thomas RK (2008). Novel MEK1 Mutation Identified by Mutational Analysis of Epidermal Growth Factor Receptor Signaling Pathway Genes in Lung Adenocarcinoma. Cancer Res.

[37] Dunford A, Weinstock DM, Savova V, Schumacher SE, Cleary JP, Yoda A, Sullivan TJ, Hess JM, Gimelbrant AA, Beroukhim R, Lawrence MS, Getz G, Lane AA (2016). Tumor-suppressor genes that escape from X-inactivation contribute to cancer sex bias. Nat Genet.

[38] van Haaften G, Dalgliesh GL, Davies H, Chen L, Bignell G, Greenman C, Edkins S, Hardy C, O'Meara S, Teague J, Butler A, Hinton J, Latimer C (2009). Somatic mutations of the histone H3K27 demethylase gene UTX in human cancer. Nat Genet.

[39] Jallades L, Baseggio L, Sujobert P, Huet S, Chabane K, Callet-Bauchu E, Verney A, Hayette S, Desvignes JP, Salgado D, Levy N, Béroud C, Felman P (2017). Exome sequencing identifies recurrent BCOR gene alterations and the absence of KLF2, TNFAIP3 and MYD88 mutations in splenic diffuse red pulp small B-cell lymphoma. Haematologica.

[40] Sengoku T, Yokoyama S (2011). Structural basis for histone H3 Lys 27 demethylation by UTX/KDM6A. Genes Dev.

[41] Ler LD, Ghosh S, Chai X, Thike AA, Heng HL, Siew EY, Dey S, Koh LK, Lim JQ, Lim WK, Myint SS, Loh JL, Ong P (2017). Loss of tumor suppressor KDM6A amplifies PRC2-regulated transcriptional repression in bladder cancer and can be targeted through inhibition of EZH2. Sci Transl Med.

[42] Van der Meulen J, Sanghvi V, Mavrakis K, Durinck K, Fang F, Matthijssens F, Rondou P, Rosen M, Pieters T, Vandenberghe P, Delabesse E, Lammens T, De Moerloose B (2015). The H3K27me3 demethylase UTX is a gender-specific tumor suppressor in T-cell acute lymphoblastic leukemia. Blood.

[43] Schuh A, Becq J, Humphray S, Alexa A, Burns A, Clifford R, Feller SM, Grocock R, Henderson S, Khrebtukova I, Kingsbury Z, Luo S, McBride D (2012). Monitoring chronic lymphocytic leukemia progression by whole genome sequencing reveals heterogeneous clonal evolution patterns. Blood.

[44] Camus V, Miloudi H, Taly A, Sola B, Jardin F (2017). XPO1 in B cell hematological malignancies: from recurrent somatic mutations to targeted therapy. J Hematol Oncol.

[45] Camus V, Stamatoullas A, Mareschal S, Viailly PJ, Sarafan-Vasseur N, Bohers E, Dubois S, Picquenot JM, Ruminy P, Maingonnat C, Bertrand P, Cornic M, Tallon-Simon V (2016). Detection and prognostic value of recurrent exportin 1 mutations in tumor and cell-free circulating DNA of patients with classical Hodgkin lymphoma. Haematologica.

[46] Jardin F, Pujals A, Pelletier L, Bohers E, Camus V, Mareschal S, Dubois S, Sola B, Ochmann M, Lemonnier F, Viailly PJ, Bertrand P, Maingonnat C (2016). Recurrent mutations of the exportin 1 gene (XPO1) and their impact on selective inhibitor of nuclear export compounds sensitivity in primary mediastinal B-cell lymphoma: XPO1 Mutations in Primary Mediastinal B-Cell Lymphoma. Am J Hematol.

[47] Jeromin S, Weissmann S, Haferlach C, Dicker F, Bayer K, Grossmann V, Alpermann T, Roller A, Kohlmann A, Haferlach T, Kern W, Schnittger S (2014). SF3B1 mutations correlated to cytogenetics and mutations in NOTCH1, FBXW7, MYD88, XPO1 and TP53 in 1160 untreated CLL patients. Leukemia.

[48] Colecchia D, Rossi M, Sasdelli F, Sanzone S, Strambi A, Chiariello M (2015). MAPK15 mediates BCR-ABL1-induced autophagy and regulates oncogene-dependent cell proliferation and tumor formation. Autophagy.

[49] Jin DH, Lee J, Kim KM, Kim S, Kim DH, Park J (2015). Overexpression of MAPK15 in gastric cancer is associated with copy number gain and contributes to the stability of c-Jun. Oncotarget.

[50] Iavarone C, Acunzo M, Carlomagno F, Catania A, Melillo RM, Carlomagno SM, Santoro M, Chiariello M (2006). Activation of the Erk8 Mitogen-activated Protein (MAP) Kinase by RET/PTC3, a Constitutively Active Form of the RET Proto-oncogene. J Biol Chem.

[51] Théry JC, Krieger S, Gaildrat P, Révillion F, Buisine MP, Killian A, Duponchel C, Rousselin A, Vaur D, Peyrat JP, Berthet P, Frebourg T, Martins A (2011). Contribution of bioinformatics predictions and functional splicing assays to the interpretation of unclassified variants of the BRCA genes. Eur J Hum Genet.

[52] Boeva V, Popova T, Lienard M, Toffoli S, Kamal M, Le Tourneau C, Gentien D, Servant N, Gestraud P, Rio Frio T, Hupe P, Barillot E, Laes JF (2014). Multi-factor data normalization enables the detection of copy number aberrations in amplicon sequencing data. Bioinformatics.

