# Supplementary material for: New generation sequencing of targeted genes in the classical and the variant form of hairy cell leukemia highlights mutations in epigenetic regulation genes
Source: Oncotarget. 2018 Jun 22;9(48):28866–76. doi: 10.18632/oncotarget.25601 (PMC6034755; doi:10.18632/oncotarget.25601)
Supplement: Supplementary file 1 [file oncotarget-09-28866-s001.pdf]

New generation sequencing of targeted genes in the classical and the variant form of hairy cell leukemia highlights mutations in epigenetic regulation genes

SUPPLEMENTARY MATERIALS

DNA and RNA purification, ARMSqPCR analysis

DNA and RNA was extracted with the automated device MagnaPur® (Roche Lifescience) according to the manufacturer's recommendations. Retro-transcription of RNA (SuperScript™ II, Invitrogen) was performed using oligodT primer (Invitrogen). gDNA and cDNA quality and quantity were measured by spectrophotometry (Nanodrop 2000®, Labtech). The presence of *BRAF*^{V600E} mutation was initially analyzed by allele specific amplification ARMSqPCR adapted from Schnittger *et al* as previously described [1].

Immunophenotyping

Multiparameter flow cytometric immunophenotype performed on a FACS CANTO II or a FACSCalibur (Becton Dickinson, (BD)) was used to characterize hairy cells (HC) and quantify tumor infiltration. PBMC (5×10^5 cells) were incubated for 30 minutes at 4° C with the following antibodies: anti-CD45-V450, -CD5-PerCpCy5,5, -CD19-PECy7, -CD23-PE, -CD43-FITC, -CD10-APC, -CD38-V450, -CD103-FITC, -CD123-PE, -CD11c-V421, -CD25-APC purchased from Becton Dickinson or anti-κ-FITC and -λ-PE from Dako. Antibody excess was washed with the lyse/lavage BD FACS® device (Becton Dickinson). Devices were standardized with EuroFlow recommendation [2]. Data were analyzed using the FACSDiva software (Becton Dickinson). Cells were gated according to side scatter-CD45$^+$, specific expression of CD19, CD11c ± CD103 ± CD123 ± CD25 and kappa/lambda restriction for measurement of tumor infiltration.

REFERENCES

1. Schnittger S, Bacher U, Haferlach T, Wendland N, Ulke M, Dicker F, Grossmann V, Haferlach C, Kern W. Development and validation of a real-time quantification assay to detect and monitor BRAFV600E mutations in hairy cell leukemia. Blood. 2012; 119: 3151–4. https://doi.org/10.1182/blood-2011-10-383323.
2. Kalina T, Flores-Montero J, van der Velden VH, Martin-Ayuso M, Böttcher S, Ritgen M, Almeida J, Lhermitte L, Asnafi V, Mendonça A, de Tute R, Cullen M, Sedek L, et al. EuroFlow standardization of flow cytometer instrument settings and immunophenotyping protocols. Leukemia. 2012; 26: 1986–2010. https://doi.org/10.1038/leu.2012.122.

A ChrX(GRCh37):g.44945109G>A,NM_021140.2(KDM6A):c.3434-1G>A (Intron 23)

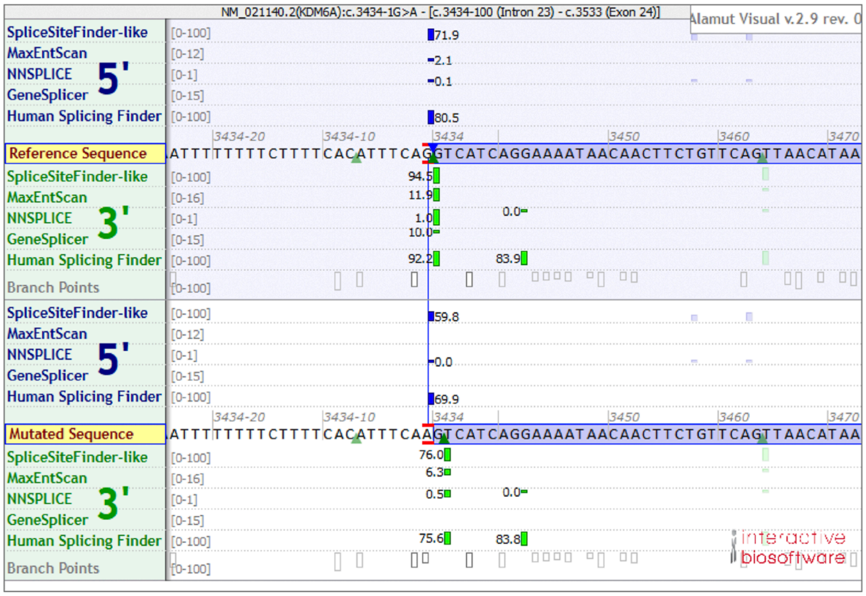

B

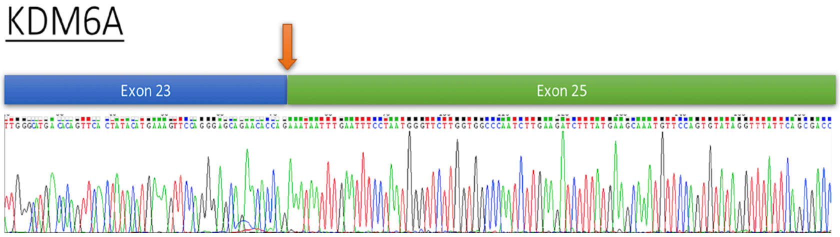

Supplementary Figure 1: Variation at 3′ splice site induces skipping of KDM6A exon 24 according to RNA sequencing. (**A**) *In silico* prediction of splicing variation for KDM6A mutation c.3434-1G > A with Alamut® Visual 2.9 (Interactive Biosoftware), diminution of prediction score of splicing site according to the mutational status. (**B**) Sanger RNA sequencing of exon 23→25 of KDM6A in UPN-v1. The mutation c.3434-1G > A (orange arrow) leads to exon 24 skipping.

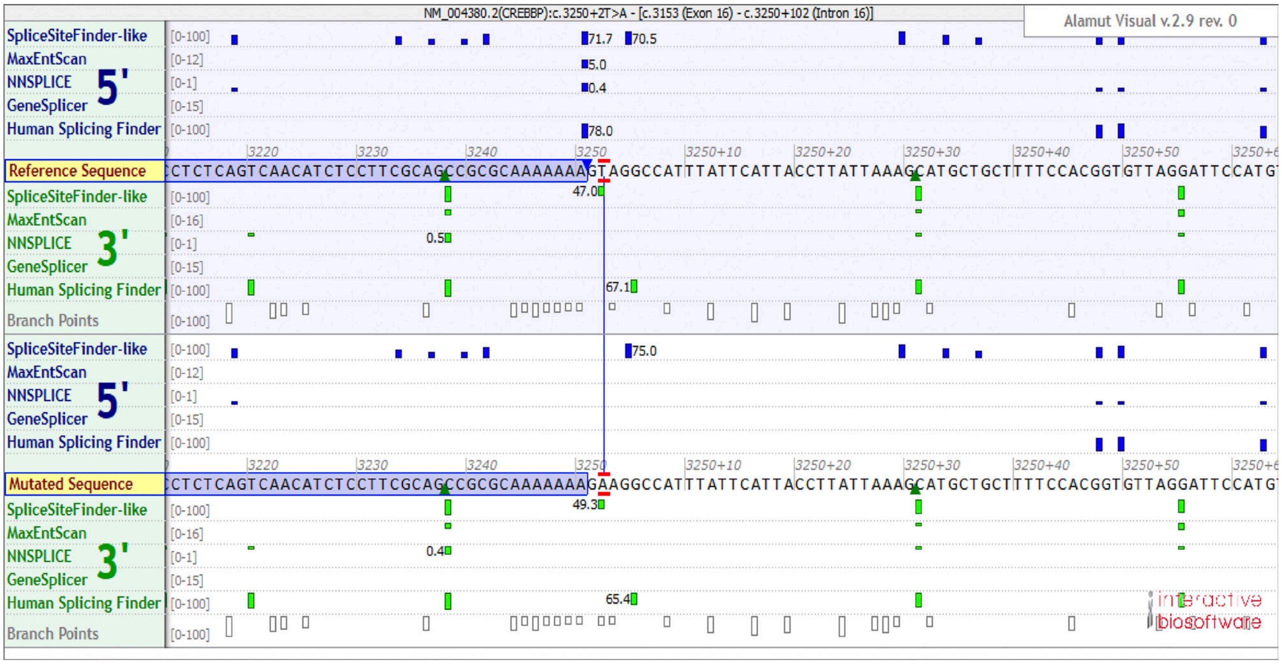

Supplementary Figure 2: Variation at 5′ splice site is predicted to induce major splicing defect of CREBBP exon 16. *In silico* prediction of slicing variation of CREBBP mutation c.3250 + 2T>A with Alamut® Visual 2.9 (Interactive Biosoftware), loss of prediction score of splicing site according to the mutational status.

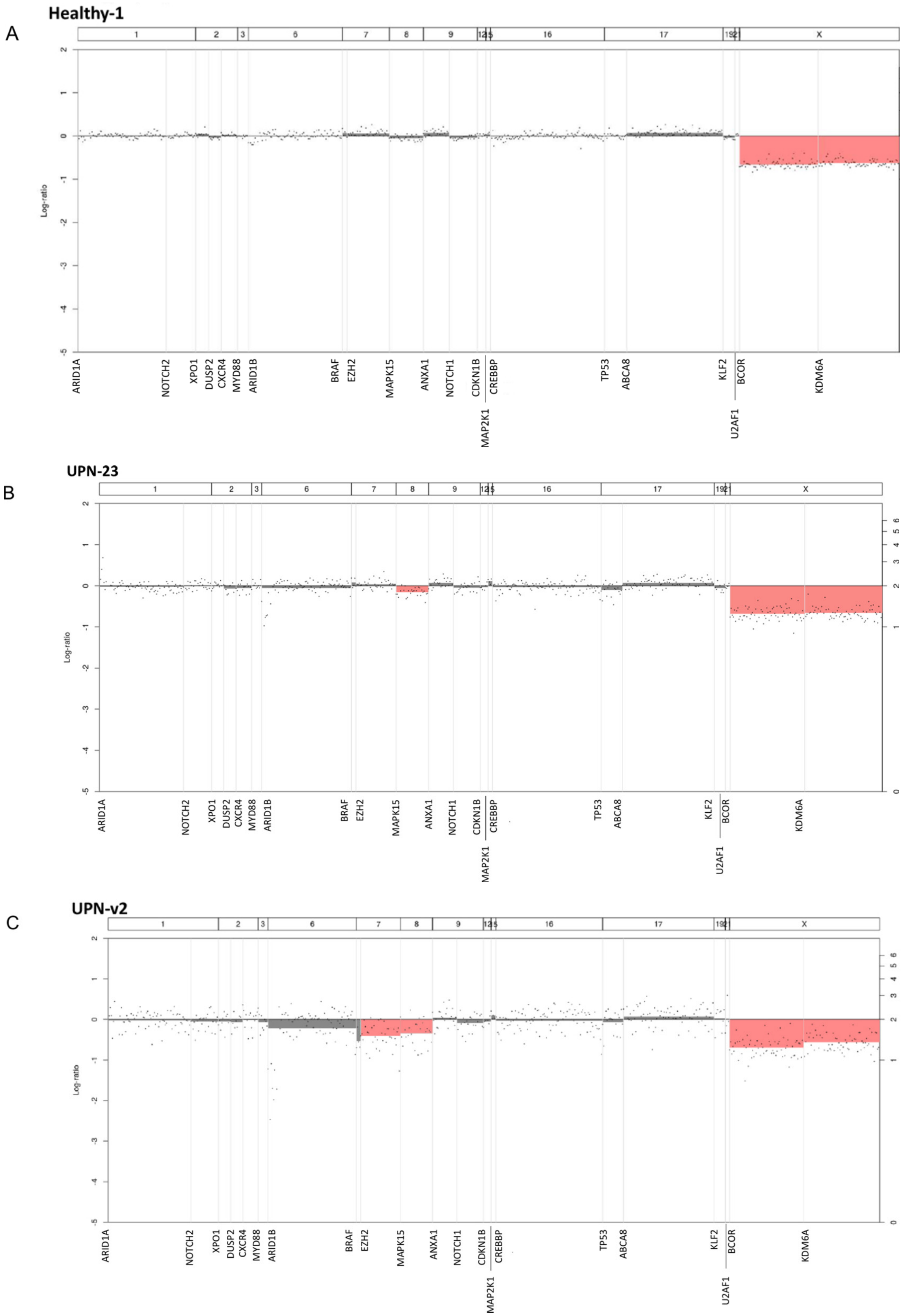

Supplementary Figure 3: Copy number evaluation Copy number (CN) variation was detected from exon sequencing of the target genes after normalization using ONCOCNV tools. (**A–C**) Log-ratio representation represent significant loss (red) and gain (blue) of copy number gene in healthy male patient (A) UPN-23 (B) and UPN-v2 (C). Statistical CNV differences was estimated with *p*-value of normalized read count and *p* values < 0.05 were considered statistically significant. (A) healthy male patient has no abnormal CNV and X monosomy was highlighted with *BCOR* and *KDM6A* loss. (B) UPN-23 have *MAPK15* loss and male phenotype (*BCOR* and *KDM6A* loss). (C) UPN-v2 have *EZH2* loss and male phenotype (*BCOR* and *KDM6A* loss). *BRAF*, *MAP2K1* and *U2AF1* CNV cannot be exploited for statistical analyze because the small number of amplicons (<5) do not allowed optimal statistical conditions.

Supplementary Table 1: Cohort data. See Supplementary_Table_1

Supplementary Table 2: Performance data of targeted sequencing

Samples	Number of mapped reads	Reads on-taget (%)	Total aligned base reads (pb)	Bases reads on taget (%)	Targeted base coverage > 20 reads (%)	Targeted base coverage > 100 reads (%)	Uniformity of base coverage (%)	Mean depth (X)
UPN-2	143341	96.13	16335463	94.14	96.82	83.76	95.58	216.6
UPN-4	135122	96.56	15360378	94.93	96.81	83.50	95.16	205.3
UPN-5	215646	90.45	23933448	90.54	97.68	93.00	95.96	305.2
UPN-6	194491	93.23	21561138	92.7	96.55	88.15	94.56	281.5
UPN-7	274949	97.22	31682354	95.24	97.65	94.26	95.17	424.9
UPN-9	59761	94.69	6755694	93.29	94.79	34.38	95.28	88.75
UPN-10	209143	94.41	23775402	92.9	97.57	90.29	94.99	311.0
UPN-11	324649	95.9	37335287	94.06	98.05	95.51	95.67	494.5
UPN-12	78689	94.76	8902188	93.34	95.87	55..08	95.09	117.0
UPN-15	222338	96.8	25250123	94.99	97.49	92.39	94.99	337.7
UPN-17	285143	96.44	31970177	95.00	97.67	94.50	95.33	427.7
UPN-18	247554	96.2	27991800	94.61	97.52	94.03	95.60	372.9
UPN-19	169486	94.6	19329437	93.06	97.23	88.24	94.58	253.3
UPN-21	231134	96.84	25954819	95.09	97.43	93.87	95.82	347.5
UPN-23	252082	96.82	28565546	95.05	97.55	93.48	94.49	382.3
UPN-24	349512	96.41	39997503	94.55	97.66	95.30	94.91	532.5
UPN-25	278394	95.74	31766338	94.06	97.78	95.87	96.38	420.8
UPN-34	219592	95.33	25317456	95.52	97.39	93.39	95.29	333.4
UPN-38	173722	87.76	19202025	88.37	97.63	86.85	95.58	238.9
UPN-40	107318	94.35	12127824	92.81	96.59	69.69	94.58	158.5
UPN-v1	284040	97.07	32410731	95.26	97.85	93.76	94.45	434.8
UPN-v2	161534	95.58	18981205	93.25	97.37	89.02	95.04	249.3
UPN-v3	228853	94.57	25647245	93.38	97.62	93.18	95.43	337.3
UPN-v4	183391	96.22	20677947	94.54	97.48	89.43	94.99	275.3
Mean	209578.5	95.17	23784647	93.78	97.25	88.08	95.21	314.46
SD	73292.57	2.17	8391393.86	1.63	0.72	13.05	0.49	112.96
Median	217619	95.82	24591785.5	94.1	97.505	93.00	95.165	322.2
Min	59761	87.76	6755694	88.37	94.79	34.38	94.45	88.75
Max	349512	97.22	39997503	95.52	98.05	95.87	96.38	532.5

Supplementary Table 3: Primers used for PCR

Gene	Exon	Primer name	Sequence (5′–3′)	Amplicon size (bp)	PCR condition
ARID1B	*1*	Forward	CACAAACTGAAAACCGTTGGC	298	60° C
	1	Reverse	CACTGTCCTTGGCGCCTC		
BCOR	*4*	Forward	CTCGTAACGGGCTCTCTCAT	245	60° C
	4	Reverse	GCAAAGGACCTGTCTACCCT		
ARID1A	*1*	Forward	GAACAATAACCTCACGGAGCC	300	60° C
	1	Reverse	TTGTACTGGTGGTTGGGGAA		
NOTCH2	*27*	Forward	GCCACCTTTCCCCTTTACAC	254	60° C
	27	Reverse	TGACATGTTCTGCCTGACCT		
ABCA8	*8*	Forward	AAGGATGCAGGAAGGTGTCT	365	60° C
	8	Reverse	ACCTACTCTGTTTGCATGTGA		
MAP2K1	*3*	Forward	GCCAATGCCTGCCTTAGTAC	298	60° C
	3	Reverse	TCGCTGTAGAACGCACCATA		
KLF2	*2*	Forward	GGGTGGTAAAAGGCAAGCAG	283	60° C
	2	Reverse	AGCAGCTCAGACACCAGG		
KLF2-1	*2*	Forward	TTTCGGTGGCCCTGGTTT	276	60° C
	2	Reverse	TGCGAACTCTTGGTGTAGGT		
KLF2-2	*2*	Forward	CTCGGGGTAGTAGAACGTGG	382	60° C
	2	Reverse	AGCAGCTCAGACACCAGG		
KLF2-3	*2*	Forward	GGTGGCCTGGTGTCTGAG	603	60° C
	2	Reverse	CAGATGCGAACTCTTGGTGT		
CDKN1B	*1*	Forward	AGTTAACCCGGGACTTGGAG	273	60° C
	1	Reverse	CCAAATGCGTGTCCTCAGAG		
KDM6A	*23*	Forward	TTTGTGCGTGTCGTATCAGC	415	60° C
	25–26	Reverse	AGGCTGTAAGTGGACCAACA		

Supplementary Table 4: SNVs identified by next generation sequencing in cell lines. See Supplementary_Table_4

Supplementary Table 5: SNVs identified by next generation sequencing in HCL-c and HCL-v. See Supplementary_Table_5
