# Supplementary material for: New generation sequencing of targeted genes in the classical and the variant form of hairy cell leukemia highlights mutations in epigenetic regulation genes
Source: Oncotarget. 2018 Jun 22;9(48):28866–76. doi: 10.18632/oncotarget.25601 (PMC6034755; doi:10.18632/oncotarget.25601)
Supplement: Supplementary file 2 [file oncotarget-09-28866-s002.docx]

Supplementary Table 1: Cohort data

| **Epidemiologic features** | | | | | | **Clinical features** | | | | | **Blood count at diagnosis** | | | | | | | **Immunophenotype** | | | | | | | | | | | | **Molecular biology** | | |
| --- | --- | --- | --- | --- | --- | --- | --- | --- | --- | --- | --- | --- | --- | --- | --- | --- | --- | --- | --- | --- | --- | --- | --- | --- | --- | --- | --- | --- | --- | --- | --- | --- |
| **UPN** | **HCL type** | **follow-up (months)** | **sex (M/F)** | **diagnosis age** | **sample** | **ttt (1/0)**** | **TFS (month)** | **Relapse (1/0)**** | **PFS (month)** | **current status** | **Hb (g/dL)** | **WB (G/L)** | **N (G/L)** | **M (G/L)** | **Ly  (G/L)** | **Thr (G/L)** | **prominent nucleolus (1/0)** | **tumor infiltration (%)** | **HCL scoring** | **CD11c** | **CD103** | **CD123** | **CD25** | **CD5** | **CD23** | **CD43** | **CD38** | **CD10** | **Light chain** | **Karyotype** | **IGHV (repertory)** | ***BRAF^V600E^* ARMS-qPCR** |
| UPN-2 | HCL-c | 103.80 | M | 45.86 | Blood | 1 | 0.56 | 0 | NA | RC | 10.6 | 3.25 | 0.39 | 0.07 | 3.43 | 17 | 0 | 60% | ≥3 | 1 | 1 | nd | 1 | 0 | 0 | nd | 0 | 1 | Kappa | nd | nd | 1 |
| UPN-4 | HCL-c | nd | F | 55.39 | Blood | nd | nd | nd | nd | nd | 12 | 1.71 | 0.37 | 0.08 | 1.24 | 53 | 0 | 31% | 4 | 1 | 1 | 1 | 1 | 1 | 1 | 0 | 1 | 0 | Kappa | nd | nd | 1 |
| UPN-5 | HCL-c | 16.49 | F | 53.62 | Blood | nd | nd | nd | nd | nd | 10.4 | 3.01 | 0.47 | 0.25 | 2.27 | 89 | 0 | 30% | 4 | 1 | 1 | 1 | 1 | 0 | 0 | 1 | 1 | 0 | Kappa | nd | nd | 1 |
| UPN-6 | HCL-c | 218.49 | M | 42.00 | Blood | 1 | 4.23 | 1 | 166.52 | RC | nd | 15 | nd | nd | 9 | nd | 0 | 96% | 4* | 1* | 1 | 1* | 1* | 0 | 0 | 0* | 0 | 0 | kappa | nd | nd | 1 |
| UPN-7 | HCL-c | 51.90 | M | 51.57 | BMA | 1 | 2.79 | 0 | NA | RC | 10.4 | 1.33 | 0.35 | 0.01 | 0.94 | 118 | 0 | 15% | 4 | 1 | 1 | 1 | 1 | 0 | 0 | 0 | 1 | 0 | Lambda | nd | nd | 1 |
| UPN-9 | HCL-c | 66.29 | M | 71.28 | BMA | 1 | 1.70 | 0 | NA | RC | 14.7 | 2.39 | 0.93 | 0.02 | 1.43 | 64 | 0 | 51% | ≥3 | 1 | 1 | nd | 1 | 0 | 0 | 0 | 0 | 1 | Lambda | nd | nd | 1 |
| UPN-10 | HCL-c | 18.03 | M | 70.84 | BMA | 1 | 1.84 | 0 | NA | RC | 13.1 | 1.72 | 0.53 | 0.02 | 1.12 | 110 | 0 | 15% | 4 | 1 | 1 | 1 | 1 | 0 | 0 | 0 | 1 | 0 | Kappa | Normal | nd | 1 |
| UPN-11 | HCL-c | 41.18 | M | 72.44 | BMA | 1 | 1.67 | 1 | 25.02 | nd | 13.8 | 2.31 | 0.69 | 0.07 | 1.46 | 99 | 0 | 33% | 4 | 1 | 1 | 1 | 1 | 0 | 0 | 0 | 0 | 0 | Lambda | nd | nd | 1 |
| UPN-12 | HCL-c | 16.23 | M | 63.93 | Blood | 1 | 1.21 | 1 | 6.20 | **DC** | 12.8 | 2.18 | 1.02 | 0.24 | 0.83 | 62 | 0 | 25% | ≥3 | 1 | 1 | nd | 1 | 0 | 0 | 1 | 0 | 0 | Lambda | nd | nd | 1 |
| UPN-15 | HCL-c | 28.79 | M | 45.68 | Blood | 1 | 6.20 | 0 | NA | RC | nd | nd | nd | nd | 25 | nd | 0 | 82% | 4 | 1 | 1 | 1 | 1 | 0 | 0 | 0 | 0 | 0 | Kappa | nd | nd | 1 |
| UPN-17 | HCL-c | 0,00 | M | 47.69 | Blood | 0 | nd | nd | 0.00 | **DC** | 6.7 | 9.8 | 0 | 0 | 9.8 | 38 | 0 | 85.5% | 4 | 1 | 1 | 1 | 1 | 0 | 0 | 0 | 0 | 0 | undetermined | nd | nd | 1 |
| UPN-18 | HCL-c | 35.74 | F | 67.73 | Blood | 1 | 30.00 | 0 | NA | RC | 13.9 | 8.3 | 4.4 | 0.16 | 3.73 | 117 | 0 | 66% | 4* | 1* | 1* | 1* | 1* | 0* | 0* | 0* | 0* | 0* | Kappa* | nd | nd | 1 |
| UPN-19 | HCL-c | 38.52 | M | 46.91 | BMA | 0 | 38.52 | 0 | NA | SD | 13.9 | 3.22 | 1.29 | 0 | 1.93 | 179 | 0 | 33.7% | 4 | 1 | 1 | 1 | 1 | 0 | 1 | 0 | 1 | 0 | Kappa | nd | nd | 1 |
| UPN-21 | HCL-c | 10.59 | F | 41.90 | BMA | 1 | 0.46 | 1 | 3.25 | RC | 12.3 | 2.83 | 1.58 | 0 | 1.44 | 54 | 0 | 40% | 4 | 1 | 1 | 1 | 1 | 0 | 1 | 0 | 0 | 0 | Kappa | Normal | nd | 1 |
| UPN-23 | HCL-c | 25.31 | M | 60.70 | Blood | 1 | 0.88 | 0 | NA | RC | 13.6 | 2.92 | 0.93 | 0 | 1.96 | 82 | 0 | 22% | 4 | 1 | 1 | 1 | 1 | 0 | 0 | 0 | 1 | 0 | kappa | nd | nd | 1 |
| UPN-24 | HCL-c | 123.44 | M | 44.8 | Blood | 1 | 0.49 | 1 | 69.08 | RC | 8.8 | 9.56 | 1.05 | 0 | 8.5 | 49 | 0 | 61% | ≥3 | 1 | 1 | nd | 1 | 0 | 0 | nd | 0 | 0 | Lambda | nd | nd | 1 |
| UPN-25 | HCL-c | 58.79 | F | 59.91 | BMA | 1 | 1.67 | 0 | NA | RC | 7.7 | 2.44 | 0.2 | 0 | 2.24 | 50 | 0 | 73% | 4 | 1 | 1 | 1 | 1 | 0 | 1 | 0 | 0 | 1 | Lambda | nd | nd | 1 |
| UPN-34 | HCL-c | nd | M | 92.38 | BMA | nd | nd | nd | nd | nd | 13.4 | 6.16 | 1.87 | 0.98 | 3.24 | 45 | 0 | 54.7% | ≥3 | 1 | 1 | nd | 1 | 0 | 0 | nd | 1 | 0 | Kappa | nd | nd | 1 |
| UPN-38 | HCL-c | 60.72 | M | 53.47 | BMA | 1 | 0.79 | 0 | NA | RC | 9.2 | 3.89 | 0.31 | 0 | 3.58 | 34 | 0 | 66% | 4 | 1 | 1 | 1 | 1 | 0 | 0 | 0 | 0 | 0 | Lambda | nd | nd | 1 |
| UPN-40 | HCL-c | 12.39 | M | 78.31 | Blood | 1 | 0.13 | 1 | 25.08 | SD | nd | nd | nd | nd | 28 | nd | 0 | 75% | 4 | 1 | 1 | 1 | 1 | 0 | 0 | 0 | 1 | 0 | Kappa | nd | nd | 0 |
| UPN-v1 | HCL-v | 19.83 | M | 64.28 | Blood | 1 | 3.15 | 1 | 4.13 | SD | 9.7 | 106 | 5.3 | 1.06 | 98.62 | 137 | 1 | 93% | 2 | 1 | 1 | 0 | 0 | 0 | 0 | 0 | 0 | 0 | Lambda | del(7q)(p2?2q3 ?6) | nd | 0 |
| UPN-v2 | HCL-v | 74.56 | M | 72.33 | BMA | 1 | 2.00 | 1 | 26.00 | RC | nd | nd | nd | nd | nd | nd | 1 | 15% | 2 | 1 | 1 | 0 | 0 | 0 | 0 | 0 | 0 | 0 | Kappa | inv(7)(p15q34) | unmutated (VH4-34) | 0 |
| UPN-v3 | HCL-v | 19.87 | M | 82.28 | Blood | 0 | 19.87 | 0 | NA | SD | 11.6 | 5.89 | 1.52 | 0.29 | 3.5 | 78 | 1 | 66% | 2 | 1 | 1 | 0* | 0 | 0 | 0 | 1 | 0 | 0 | Kappa | nd | nd | 0 |
| UPN-v4 | HCL-v | 8.95 | M | 67.08 | Blood | 0 | 8.95 | 0 | NA | SD | 16.1 | 16.12 | 7.42 | 0.48 | 8.22 | 162 | 1 | 74.9% | 1 | 1 | 0 | 0 | 0 | 0 | 0 | 0 | 0 | 0 | Lambda | nd | nd | 0 |

** 1 corresponds to positive. 0 corresponds to negative; * markers tested on another sample. HCL scoring based on Matutes et al. 1994 (16)

Abbreviations: BMA. bone marrow aspirate; M. Male; F. Female; ttt. treatment; TFS. Treatment Free Survival; PFS. Progression Free Survival; CR. complete response; SD. stable disease; DC. death; Hb. hemoglobin; WB. leucocyte; N. neutrophil; M. monocyte; Ly. lymphocyte; Thr. thrombocyte; HC. hairy cell.
