# Supplementary material for: New generation sequencing of targeted genes in the classical and the variant form of hairy cell leukemia highlights mutations in epigenetic regulation genes
Source: Oncotarget. 2018 Jun 22;9(48):28866–76. doi: 10.18632/oncotarget.25601 (PMC6034755; doi:10.18632/oncotarget.25601)
Supplement: Supplementary file 3 [file oncotarget-09-28866-s003.docx]

Supplementary Table 4: SNVs identified by next generation sequencing in cell lines

| **Cell line** | **Gene** | **Chr** | **Position** | **Infiltration/samples (%)** | **VAF (%)** | **rVAF** | **Chromosome number variation*** | **Depth per amplicon** | **NM_code** | **DNA change** | **Protein Change** | **Mutation type** | **CADD result** | **SIFT result** | **Polyphen-2 result** | **Splicing variant prediction ALAMUT result** | **COSMIC database** |
| --- | --- | --- | --- | --- | --- | --- | --- | --- | --- | --- | --- | --- | --- | --- | --- | --- | --- |
| BONNA-12 | *NOTCH1* | 9 | 139390794 | 100 | 32.29 | 0.32 | Partial Trisomy of chr 9 | 628 | NM_017617 | c.C7397T | p.T2466M | nonsynonymous SNV | benign | damaging | damaging | NA | [COSM12778](http://cancer.sanger.ac.uk/cosmic/mutation/overview?id=12778) |
| JOK-1 | *TP53* | 17 | 7577581 | 100 | 48.11 | 0.48 | Euploïdy of chr 17 | 433 | NM_000546 | c.T700C | p.Y234H | nonsynonymous SNV | damaging | damaging | damaging | NA | [COSM238605](http://cancer.sanger.ac.uk/cosmic/mutation/overview?id=238605) |
| JOK-1 | *TP53* | 17 | 7578211 | 100 | 53.72 | 0.54 | Euploïdy of chr 17 | 289 | NM_000546 | c.G638A | p.R213Q | nonsynonymous SNV | damaging | damaging | damaging | NA | [COSM10735](http://cancer.sanger.ac.uk/cosmic/mutation/overview?id=10735) |
| JVM-3 | *ARID1B* | 6 | 6157522032 | 100 | 25.28 | 0.25 | Tetraploïdy of chr 6 | 334 | NM_020732 | c.C4304T | p.P1435L | nonsynonymous SNV | benign | damaging | possibly damaging | NA | [COSM1183556](http://cancer.sanger.ac.uk/cosmic/mutation/overview?id=1183556) |
| JVM-3 | *BRAF* | 7 | 140453132 | 100 | 49.54 | 0.50 | Tetraploïdy of chr 7 with del(7q)x2 | 320 | NM_004333 | c.A1803T | p.K601N | nonsynonymous SNV | damaging | damaging | damaging | NA | [COSM6265](http://cancer.sanger.ac.uk/cosmic/mutation/overview?id=6265) |

* Chromosome number variation analyzed by karyotyping

NA: not applicable
