# Supplementary material for: New generation sequencing of targeted genes in the classical and the variant form of hairy cell leukemia highlights mutations in epigenetic regulation genes
Source: Oncotarget. 2018 Jun 22;9(48):28866–76. doi: 10.18632/oncotarget.25601 (PMC6034755; doi:10.18632/oncotarget.25601)
Supplement: Supplementary file 4 [file oncotarget-09-28866-s004.docx]

Supplementary Table 5: SNVs identified by next generation sequencing in HCL-c and HCL-v

| **Sample** | **Gene** | **Chr** | **Position** | **Infiltration/sample* (%)** | **VAF (%)** | **rVAF** | **Depth per amplicons** | **NM_code** | **DNA change** | **Protein change** | **Mutation type** | **CADD result** | **SIFT result** | **Polyphen2 result** | **Splicing variant prediction ALAMUT result** | **COSMIC database or reference** |
| --- | --- | --- | --- | --- | --- | --- | --- | --- | --- | --- | --- | --- | --- | --- | --- | --- |
| UPN-2 | BRAF | 7 | 140453136 | 60 | 33.33 | 0.56 | 159 | NM_004333 | c.T1799A | p.V600E | nonsynonymous SNV | damaging | damaging | damaging | NA | [COSM476](http://cancer.sanger.ac.uk/cosmic/mutation/overview?id=476) |
| UPN-2 | CDKN1B | 12 | 12871879A | 60 | 34.78 | 0.58 | 253 | NM_004064 | c.A596C | p.X199S | Stoploss SNV | nd | damaging | nd | NA | [COSM4956997](http://cancer.sanger.ac.uk/cosmic/mutation/overview?id=4969597) |
| UPN-4 | BRAF | 7 | 140453136 | 31 | 22.22 | 0.72 | 207 | NM_004333 | c.T1799A | p.V600E | nonsynonymous SNV | damaging | damaging | damaging | NA | [COSM476](http://cancer.sanger.ac.uk/cosmic/mutation/overview?id=476) |
| UPN-5 | BRAF | 7 | 140453136 | 30 | 17.2 | 0.57 | 250 | NM_004333 | c.T1799A | p.V600E | nonsynonymous SNV | damaging | damaging | damaging | NA | [COSM476](http://cancer.sanger.ac.uk/cosmic/mutation/overview?id=476) |
| UPN-6 | BRAF | 7 | 140453136 | 96 | 35.96 | 0.37 | 228 | NM_004333 | c.T1799A | p.V600E | nonsynonymous SNV | damaging | damaging | damaging | NA | [COSM476](http://cancer.sanger.ac.uk/cosmic/mutation/overview?id=476) |
| UPN-7 | BRAF | 7 | 140453136 | 15 | 5.52 | 0.37 | 362 | NM_004333 | c.T1799A | p.V600E | nonsynonymous SNV | damaging | damaging | damaging | NA | [COSM476](http://cancer.sanger.ac.uk/cosmic/mutation/overview?id=476) |
| UPN-9 | BRAF | 7 | 140453136 | 51 | 17.14 | 0.34 | 70 | NM_004333 | c.T1799A | p.V600E | nonsynonymous SNV | damaging | damaging | damaging | NA | [COSM476](http://cancer.sanger.ac.uk/cosmic/mutation/overview?id=476) |
| UPN-11 | BRAF | 7 | 140453136 | 33 | 16.96 | 0.51 | 448 | NM_004333 | c.T1799A | p.V600E | nonsynonymous SNV | damaging | damaging | damaging | NA | [COSM476](http://cancer.sanger.ac.uk/cosmic/mutation/overview?id=476) |
| UPN-11 | KLF2 | 19 | 16436775 | 33 | 17.11 | 0.52 | 561 | NM_016270 | c.G824A | p.S275N | nonsynonymous SNV | damaging | tolerated | damaging | NA | Piva et al 2015 (25) |
| UPN-12 | BRAF | 7 | 140453136 | 25 | 14.55 | 0.58 | 110 | NM_004333 | c.T1799A | p.V600E | nonsynonymous SNV | damaging | damaging | damaging | NA | [COSM476](http://cancer.sanger.ac.uk/cosmic/mutation/overview?id=476) |
| UPN-15 | BRAF | 7 | 140453136 | 82 | 44.65 | 0.54 | 271 | NM_004333 | c.T1799A | p.V600E | nonsynonymous SNV | damaging | damaging | damaging | NA | [COSM476](http://cancer.sanger.ac.uk/cosmic/mutation/overview?id=476) |
| UPN-17 | BRAF | 7 | 140453136 | 86 | 39.74 | 0.46 | 307 | NM_004333 | c.T1799A | p.V600E | nonsynonymous SNV | damaging | damaging | damaging | NA | [COSM476](http://cancer.sanger.ac.uk/cosmic/mutation/overview?id=476) |
| UPN-18 | BRAF | 7 | 140453136 | 66 | 72.39 | 1.10 | 297 | NM_004333 | c.T1799A | p.V600E | nonsynonymous SNV | damaging | damaging | damaging | NA | [COSM476](http://cancer.sanger.ac.uk/cosmic/mutation/overview?id=476) |
| UPN-18 | ARID1B | 6 | 157511267 | 66 | 44.58 | 0.68 | 1521 | NM_020732 | c.A3785C | p.Q1262P | nonsynonymous SNV | damaging | tolerated | benign | NA | NA |
| UPN-18 | CREBBP | 16 | 3900685 | 66 | 17.15 | 0.26 | 1009 | NM_004380 | c.401_411del | p.134_137del | frameshift deletion | nd | nd | nd | NA | NA |
| UPN-19 | BRAF | 7 | 140453136 | 34 | 15.92 | 0.47 | 245 | NM_004333 | c.T1799A | p.V600E | nonsynonymous SNV | damaging | damaging | damaging | NA | [COSM476](http://cancer.sanger.ac.uk/cosmic/mutation/overview?id=476) |
| UPN-21 | BRAF | 7 | 140453136 | 40 | 11.37 | 0.28 | 255 | NM_004333 | c.T1799A | p.V600E | nonsynonymous SNV | damaging | damaging | damaging | NA | [COSM476](http://cancer.sanger.ac.uk/cosmic/mutation/overview?id=476) |
| UPN-23 | BRAF | 7 | 140453136 | 22 | 11.93 | 0.54 | 285 | NM_004333 | c.T1799A | p.V600E | nonsynonymous SNV | damaging | damaging | damaging | NA | [COSM476](http://cancer.sanger.ac.uk/cosmic/mutation/overview?id=476) |
| UPN-23 | KLF2 | 19 | 16436775 | 22 | 10.49 | 0.48 | 286 | NM_016270 | c.G824C | p.S275T | nonsynonymous SNV | benign | tolerated | benign | NA | [COSM335797](http://cancer.sanger.ac.uk/cosmic/mutation/overview?id=3357097) |
| UPN-24 | BRAF | 7 | 140453136 | 61 | 27.78 | 0.46 | 396 | NM_004333 | c.T1799A | p.V600E | nonsynonymous SNV | damaging | damaging | damaging | NA | [COSM476](http://cancer.sanger.ac.uk/cosmic/mutation/overview?id=476) |
| UPN-25 | BRAF | 7 | 140453136 | 73 | 42.94 | 0.59 | 361 | NM_004333 | c.T1799A | p.V600E | nonsynonymous SNV | damaging | damaging | damaging | NA | [COSM476](http://cancer.sanger.ac.uk/cosmic/mutation/overview?id=476) |
| UPN-25 | CDKN1B | 12 | 12871001 | 73 | 6.51 | 0.09 | 169 | NM_004064 | c.G228A | p.W76X | stopgain SNV | damaging | tolerated | nd | NA | [COSM1628449](http://cancer.sanger.ac.uk/cosmic/mutation/overview?id=1628449) |
| UPN-25 | NOTCH1 | 9 | 139391806 | 73 | 46.58 | 0.64 | 307 | NM_017617 | c.C6385G | p.L2129V | nonsynonymous SNV | begnin | tolerated | damaging | NA | NA |
| UPN-34 | BRAF | 7 | 140453136 | 55 | 17.24 | 0.31 | 261 | NM_004333 | c.T1799A | p.V600E | nonsynonymous SNV | damaging | damaging | damaging | NA | [COSM476](http://cancer.sanger.ac.uk/cosmic/mutation/overview?id=476) |
| UPN-38 | BRAF | 7 | 140453136 | 66 | 31.47 | 0.48 | 197 | NM_004333 | c.T1799A | p.V600E | nonsynonymous SNV | damaging | damaging | damaging | NA | [COSM476](http://cancer.sanger.ac.uk/cosmic/mutation/overview?id=476) |
| UPN-38 | KLF2 | 19 | 16436763 | 66 | 27.68 | 0.42 | 289 | NM_016270 | c.C812T | p.T271I | nonsynonymous SNV | damaging | tolerated | possibly damaging | NA | Piva et al 2015 (25) |
| UPN-38 | KLF2 | 19 | 16436802 | 66 | 10.42 | 0.16 | 288 | NM_016270 | c.C851G | p.T284S | nonsynonymous SNV | damaging | tolerated | possibly damaging | NA | Piva et al 2015 (25) |
| UPN-40 | ARID1A | 1 | 27059264 | 75 | 38.62 | 0.51 | 145 | NM_006015 | c.C1901G | p.S634X | stopgain SNV | damaging | tolerated | nd | NA | [COSM1296222](http://cancer.sanger.ac.uk/cosmic/mutation/overview?id=1296222) |
| UPN-40 | MAP2K1 | 15 | 66727455 | 75 | 39.19 | 0.52 | 74 | NM_002755 | c.G171C | p.K57N | nonsynonymous SNV | damaging | tolerated | damaging | NA | [COSM5520914](http://cancer.sanger.ac.uk/cosmic/mutation/overview?id=5520914) |
| UPN-v1 | KDM6A | X | 44945109 | 95 | 93.82 | 0.99 | 178 | NM_021140 | c.3434-1G>A | splicing variant | splicing variant | NA | NA | NA | damaging | [COSM4411954](http://cancer.sanger.ac.uk/cosmic/mutation/overview?id=4411954) |
| UPN-v1 | MAP2K1 | 15 | 66729100 | 95 | 6.18 | 0.07 | 534 | NM_002755 | c.T308A | p.I103N | nonsynonymous SNV | damaging | damaging | damaging | NA | [COSM3728157](http://cancer.sanger.ac.uk/cosmic/mutation/overview?id=3728157) |
| UPN-v2 | KDM6A | X | 44918613 | 15 | 18.24 | 1.22 | 141 | NM_021140 | c.1096delA | p.N366fs | frameshift deletion | nd | nd | nd | NA | NA |
| UPN-v3 | CREBBP | 16 | 3817719 | 66 | 22.5 | 0.34 | 551 | NM_004380 | c.3261+2T>A | splicing variant | splicing variant | NA | NA | NA | damaging | NA |
| UPN-v3 | MAP2K1 | 15 | 66727451 | 66 | 31.71 | 0.48 | 328 | NM_002755 | c.A167C | p.Q56P | nonsynonymous SNV | damaging | tolerated | damaging | NA | [COSM1235481](http://cancer.sanger.ac.uk/cosmic/mutation/overview?id=1235481) |
| UPN-v4 | ARID1A | 1 | 27023808 | 75 | 56.16 | 0.75 | 203 | NM_006015 | c.C914T | p.A305V | nonsynonymous SNV | damaging | tolerated | possibly damaging | NA | NA |
| UPN-6-R1 | BRAF | 7 | 140453136 | 50 | 32.16 | 0.64 | 569 | NM_004333 | c.T1799A | p.V600E | nonsynonymous SNV | damaging | damaging | damaging | NA | [COSM476](http://cancer.sanger.ac.uk/cosmic/mutation/overview?id=476) |
| UPN-40-R2 | ARID1A | 1 | 27059264 | 78 | 38.75 | 0.50 | 542 | NM_006015 | c.C1901G | p.S634X | stopgain SNV | damaging | tolerated | nd | NA | [COSM1296222](http://cancer.sanger.ac.uk/cosmic/mutation/overview?id=1296222) |
| UPN-40-R2 | MAP2K1 | 15 | 66727455 | 78 | 31.88 | 0.41 | 276 | NM_002755 | c.G171C | p.K57N | nonsynonymous SNV | damaging | tolerated | damaging | NA | [COSM5520914](http://cancer.sanger.ac.uk/cosmic/mutation/overview?id=5520914) |
| UPN-40-R2 | BCOR | X | 39921430 | 78 | 11.8 | 0.15 | 466 | NM_001123384 | c.G4234T | p.E1412X | stopgain SNV | damaging | damaging | nd | NA | NA |
| UPN-40-R2 | XPO1 | 2 | 61719472 | 78 | 1.93 | 0.02 | 519 | NM_003400 | c.G1711A | p.E571K | nonsynonymous SNV | damaging | damaging | damaging | NA | [COSM96797](http://cancer.sanger.ac.uk/cosmic/mutation/overview?id=96797) |
| UPN-v1-R2 | KDM6A | X | 44945109 | 88 | 86.57 | 0.98 | 283 | NM_021140 | c.3434-1G>A | splicing variant | splicing variant | NA | NA | NA | damaging | [COSM4411954](http://cancer.sanger.ac.uk/cosmic/mutation/overview?id=4411954) |
| UPN-v1-R2 | MAP2K1 | 15 | 66729100 | 88 | 36.36 | 0.41 | 715 | NM_002755 | c.T308A | p.I103N | nonsynonymous SNV | damaging | damaging | damaging | NA | [COSM3728157](http://cancer.sanger.ac.uk/cosmic/mutation/overview?id=3728157) |
| UPN-v2-R1 | KDM6A | X | 44918613 | 83 | 83.69 | 1.01 | 141 | NM_021140 | c.1096delA | p.N366fs | frameshift deletion | nd | nd | nd | NA | NA |
| UPN-v3-E | CREBBP | 16 | 3817719 | 55 | 3.26 | 0.06 | 522 | NM_004380 | c.3261+2T>A | splicing variant | splicing variant | NA | NA | NA | damaging | NA |
| UPN-v3-E | MAP2K1 | 15 | 66727451 | 55 | 51.25 | 0.93 | 400 | NM_002755 | c.A167C | p.Q56P | nonsynonymous SNV | damaging | tolerated | damaging | NA | [COSM1235481](http://cancer.sanger.ac.uk/cosmic/mutation/overview?id=1235481) |

* Tumor infiltration was determined by FCM

NA, not applicable; nd, not done.
